# The Regulation of microRNAs in Alzheimer's Disease

**DOI:** 10.3389/fneur.2020.00288

**Published:** 2020-04-17

**Authors:** Xianjuan Kou, Dandan Chen, Ning Chen

**Affiliations:** Hubei Key Laboratory of Exercise Training and Monitoring, Tianjiu Research and Development Center for Exercise Nutrition and Foods, College of Health Science, Wuhan Sports University, Wuhan, China

**Keywords:** Alzheimer's disease, Aβ, microRNA, biomarker, autophagy

## Abstract

MicroRNAs are small non-coding nucleic acids that are responsible for regulating the gene expression by binding to the coding region and 3' and 5' un-translated region of target messenger RNA. Approximately 70% of known microRNAs are expressed in the brain and increasing evidences demonstrate the possible involvement of microRNAs in Alzheimer's disease (AD) according to the statistics. The characteristic symptoms of AD are the progressive loss of memory and cognitive functions due to the deposition of amyloid β (Aβ) peptide, intracellular aggregation of hyperphosphorylated Tau protein, the loss of synapses, and neuroinflammation, as well as dysfunctional autophagy. Therefore, microRNA-mediated regulation for above-mentioned changes may be the potential therapeutic strategies for AD. In this review, the role of specific microRNAs involved in AD and corresponding applications are systematically discussed, including positive effects associated with the reduction of Aβ or Tau protein, the protection of synapses, the inhibition of neuroinflammation, the mitigation of aging, and the induction of autophagy in AD. It will be beneficial to develop effective targets for establishing a cross link between pharmacological intervention and AD in the near future.

## Introduction

Alzheimer's disease (AD) is the most common form of late-life dementia, with the characteristics of memory loss, impaired cognitive function, and various neuropsychiatric disturbances. The pathological changes in AD include the deposition of amyloid β (Aβ) peptide as senile plaques (SPs), the aggregation of hyperphosphorylated Tau protein as neurofibrillary tangles (NFTs), and neurodegeneration. With the acceleration of an aging society, the more and more elderly will be suffered from AD in the future. Although the underlying molecular mechanisms of AD are still unknown, the growing evidence indicates that the deposition of Aβ, the abnormal aggregation of Tau protein, and neuroinflammation play major roles in the pathogenesis of AD.

MicroRNAs with the length of 18–25 nucleotides, as the post-transcriptional regulators of gene expression, usually down-regulate the expression of mRNA and protein upon targeting specific mRNAs by binding to (3′-UTR) of the targets. It has been estimated that about 60% of human genes are regulated by microRNAs (1–3), suggesting that these microRNAs play critical roles in a series of biological processes. Data from literature have shown that microRNAs are widely found in the nervous system, some microRNAs exhibit the abundant expression in the brain and participate in neuronal development, synaptic plasticity, neuronal differentiation, and the pathogenesis of neurodegenerative disorders (4). Meanwhile, the expression of some microRNAs is dynamically regulated during the process of brain development, neuronal maturation and neurogenesis (5). Therefore, the slight aberration in the expression and activity of microRNAs may be detrimental to brain functions (6–8).

Although pathological features of AD are very well-documented; unfortunately, the available treatments cannot terminate disease progression, but can slow down it. Recently, growing evidence has demonstrated that the dysfunction of microRNAs within neurons and the altered expression of microRNAs are highly associated with the pathogenesis of neurodegenerative diseases (9–12). Thus, the regulation of microRNAs by exogenous interventions will provide a new perspective to explore the pathogenesis and neuropathology of AD.

## microRNAs Involvement in AD

### Aβ Regulation Mediated by microRNAs in AD

Excessive accumulation of Aβ may induce significant cytotoxicity in neurons, and is a key pathogenic factor of AD. Increasing evidence suggests that microRNAs can affect Aβ production. Several studies have used profiling strategies to identify the dys-regulation of microRNAs in AD. Some dys-regulated microRNAs involved in the regulation of Aβ deposition have been reported in human brain, mouse models, and cell lines with AD (9, 13). Aβ peptide is produced from amyloid precursor protein (APP) after cleaved by beta-site APP cleaving enzyme 1 (BACE1). Moreover, presenilin 1 (PS1) mutation with the function to produce Aβ from its precursor beta APP can enhance p53 activity in human embryonic kidney (HEK)-293 cells and p53 expression in familial AD (FAD)-affected brains (14). Some specific microRNAs have been reported to be either up-regulated or down-regulated in AD. For example, miR-9, miR-29, miR-29a/b-1, miR-124, miR-101, miR-107, miR-298, and miR-328 contribute to the increase of Aβ production, all of them can exhibit the reduced expression in patients or model animals with AD by regulating the expression of BACE1 and/or APP (15–18). Data from clinical studies have demonstrated that miR-29a/101 in peripheral whole blood from AD patients is markedly down-regulated (19). Certain microRNAs also participate in physiological regulation of APP levels. For example, the overexpression of miR-106a and miR-520c can result in the significant reduction of APP level in HEK-293 cells (20). Moreover, one study has demonstrated that the reduced expression of miR-16 can potentially cause the accumulation of APP protein in the embryo of spontaneous senescence-accelerated mouse P8 (SAMP8) model mice with AD; in contrast, the overexpression of miR-16 also can cause the decreased expression of APP protein *in vitro* and *in vivo* (17). Thus, the exogenous overexpression of these microRNAs may play a critical role in the generation of Aβ. The overexpression of miR-29 in humans and transgenic mice can cause the decrease of endogenous BACE1 and the increase of Aβ production (9). Meanwhile, the decreased expression of miR-17, miR-101 and miR-16 is accompanied with high APP level (21), suggesting that the overexpression of miR-17, miR-101 and miR-16 suppresses APP. Another class of microRNAs down-regulated in 12-month-old SAMP8 mice is miR-195 when compared with SAMR1 mice (22). The overexpression of miR-195 in N2a/APP695 cells presents the decreased Aβ level, while the inhibition of miR-195 leads to the increase of Aβ. The reduced expression of these microRNAs may result in the elevated expression and function of BACE1, thus causing aberrant Aβ production as the characteristics of the brains from humans and mice with AD. In addition, overexpressed miR-186 in neuronal cells can result in reduced Aβ level by suppressing BACE1 expression; however, the down-regulated endogenous miR-186 can cause the increased BACE1 level (23). These findings provide the molecular mechanisms associated with BACE1, APP and Aβ deregulation in AD and new perspectives for the etiology of this disease. However, it remains unclear whether the reduced microRNAs play a primary role in the induction of AD. Besides, other microRNAs increase Aβ levels; for example, miR-128 is involved in the development and progression of AD. The levels of miR-128 and Aβ are significantly increased in the cerebral cortex of 3xTg-AD mice when compared with wild type mice; in contrast, miR-128 knockout mice reveal the improvement of cognitive capacity when compared with 3xTg-AD mice. In another study (24), the inhibition of miR-126 has been found to be neuroprotective against Aβ42 toxicity, suggesting that both miR-128 and miR-126 may be the important mechanistic link with AD progression (25).

### The microRNA-Medicated Hyperphosphorylation of Tau Protein in AD

In addition to Aβ, the accumulation of intracellular insoluble hyperphosphorylated Tau protein is another pathological feature in AD. The detrimental effects of altered microRNAs in AD neurons may not be restricted to Aβ production and deposition. MicroRNA is also closely related to the phosphorylation and pathological aggregation of Tau protein. For example, miR-132 has a strong regulatory effect on the central nervous system. According to the studies on miR-132/-212 double knockout mouse model, double knockout mice exhibit significant cognitive deficits in recognition, new object recognition and spatial memory (26). In addition, miR-132/-212 has been reported as the down-regulation in the frontal cortex of the AD subjects with mild cognitive decline (27), thus confirming that miR-132/-212 plays a critical regulatory role in cognitive capacity. On the other hand, miR-101b mimic can rescue Tau pathology, dendritic abnormality, and memory deficits in AD mice (28). MiR-137 level has been shown to be a regulator of neuronal development and cognitive function; and clinically to be decreased in the serum of patients with AD so that it could be used as a marker for early diagnosis (29). Similarly, the level of miR-137 also exhibits a decrease in APP/PS1 transgenic mice; however, miR-137 mimics can inhibit p-Tau (Ser202, Ser396, and Ser404) induced by Aβ1-42 in SH-SY5Y cells. In addition, miR-15a, as one of the members in miR-15 family, is frequently down-regulated in AD (30). Moreover, miR-15a can target extracellular signal-regulated kinase 1 (ERK1) for the involvement of Tau hyperphosphorylation (9). The decreased miR-15 can participate neuronal Tau hyperphosphorylation. Data from clinical trials indicate that miR-106b is down-regulated in sporadic AD patients and SH-SY5Y cells (31), and can inhibit Aβ42-induced Tau phosphorylation at the site of Tyr18. Similarly, the expression of miR-512 from Tau protein-rich brains of the patients with advanced AD is significantly reduced, indicating that miR-512 can negatively regulate Tau protein through targeting Fas-related death domain protein (32). Furthermore, miR-153 from the frontal cortex of AD patients is reduced when compared with age-matched control (33). Of course, there are some abnormally elevated microRNAs involved in the hyperphosphorylation of Tau protein, and miR-125b is markedly elevated in animal models with AD. In C57BL/6 wild-type mice, the injection with miR-125b can cause increased phosphorylation of Tau protein and impaired learning and memory capacity (34). Similarly, in primary hippocampal neurons, the overexpression of miR-125b can lead to Tau hyperphosphorylation, affect synaptic morphology, and accelerate apoptosis (34, 35). Conversely, the suppression of miR-125b in primary neurons can reduce Tau phosphorylation and kinase expression/activity. There is inconsistent with the protective role of miR-125b in AD. In IL-1β-induced primary co-culture of human neuronal-glial cells, miR-146a is significantly up-regulated. The expression of miR-146a correlated with senile plaque density and synaptic pathology in the Tg2576 and 5xFAD TG mouse models (36). In China, based on the study involved in 52 patients with mild and moderate AD, the treatment with modified Shuyu Pill could effectively improve the cognitive function of the patients with mild and moderate AD and the underlying mechanism may be related to inhibiting the expressions of IL-1β/NF-κB/miR-146a in peripheral blood (37). In addition, the up-regulation of miR-26b in temporal cortex of the AD models from the early prodromal stage, and the elevated level of miR-26b in postmitotic rodent and human neurons can contribute to the phosphorylation of Tau and apoptotic cell death (38). Another study has shown that compared with the normal elderly group, miR-34a in blood mononuclear cells of AD patients is significantly down-regulated for regulating the phosphorylation of Tau (39), suggesting that miR-34a could be used as a non-invasive biomarker for AD. Similarly, the co-aggregation of Tau could be associated with specific mutations of PSEN1 and/or PSEN2 genes in sporadic and dominantly inherited AD (40).

### microRNA-Mediated Synaptic Dysfunction in AD

The alteration in synaptic plasticity is one of the important features for patients with AD. The genome-wide transcriptome studies indicate that many key genes for synapse activity are down-regulated in AD (41). The recovery of cognitive function can be achieved by restoring the reduced microRNAs acting at the synaptic level. The abnormal down-regulation of miR-188-5p is reported in the cerebral cortices and hippocampus of AD patients when compared with age-matched control subjects (42). Dendritic spine and synapse loss are well-documented in AD. However, the overexpression of miR-188-5p alleviates the decrease in dendritic spine density in rat primary hippocampal neuron cultures with the exposure of Aβ. Long-term potentiation (LTP) is believed to be a synaptic mechanism underlying the storage of long-term memory in the brain. The replenishment of miR-188-5p can improve behavioral outcomes and enhance synaptic activity, importantly, and restore cognitive function in AD mouse models such as 5XFAD mice (42, 43). However, some microRNAs are abnormally elevated in AD models and could have negative effects on neurons. Thus, they could need to be down-regulated by exogenous means. In the study of AD patients, miR-34a/p73 expression is found to be remarkably increased in hippocampal tissues, which participates in modulating synaptic activity by lessening synaptotagmin-1 expression (44). Through microRNA microarray screening analysis, the significant up-regulation of miR-30b in the brain of AD patients and transgenic mice is observed (45). The overexpression of miR-30b in hippocampal tissues can jeopardize synaptic structure and function of hippocampal neurons; in turn, can cause the deficits in cognitive function in normal wild type animals. In contrast, the knockdown of miR-30b in transgenic mice prevents synaptic and cognitive decline. These findings suggest that memory deficits in AD may be caused by microRNA alterations. Additionally, the up-regulation of miR-181 and SIRT1 and the decreased c-Fos protein level are observed in the dorsal and ventral hippocampal tissues of 3xTg-AD mice. SIRT1 and c-Fos transcription factor are involved in memory consolidation as the potential targets of miR-181 (46). Another study has reported that Aβ induces the up-regulation of miR-124 in the brain of Tg2576 mice (47). PTPN1 has been implicated in the formation of hippocampal synapses and learning capacity. Importantly, miR-124 directly targets the 3′-UTR of PTPN1 to suppress its translation, thus disrupting synaptic transmission, plasticity and memory. Consistent with these findings, the up-regulation of miR-574 in APP/PSEN1 mice has been reported and miR-574-5p can influence the expression of neuritin (NRN1) involved in synaptic plasticity (48). The results from HT22 hippocampal neuronal cells have shown that miR-574 inhibitors significantly promote NRN1 expression. Additionally, the treatment with Aβ42 can cause the increase of miR-142-5p in SH-SY5Y neuronal cells. In contrast, the inhibition of miR-142-5p can rescue Aβ42-mediated synaptic dysfunction (49). These findings suggest that the reversal of dys-regulated miR-30b, miR-124, miR-574-5p, and miR-142-5p in the brain may prevent or slow cognitive decline in AD. BDNF as a neurotrophic factor plays a pivotal role in synaptic plasticity and cognition. Previous studies have demonstrated that a reduction in BDNF within the prefrontal cortex and hippocampus is highly related to cognitive deficits in animal models with AD (50, 51). Recent study has demonstrated that miR-10a is also a negative regulator in synapse remodeling as a result of the reduction in BDNF-TrkB signals in AD rats. Similarly, the up-regulation of miR-206 in hippocampal tissue, cerebrospinal fluid, and plasma of APP/PSEN1 transgenic mice is also observed, and the alteration of miR-206 contributes to the pathology of AD through down-regulating BDNF (52).

### The Modulation Role of Neuroinflammation in AD

Neuroinflammation in brain tissues of AD models is primarily mediated by microglia and astrocytes. It is a high risk of AD and involved in the pathological process of AD. This is substantiated by increased levels of pro-inflammatory cytokines including TNF-α and/or IL-6 in serum and brain tissue of AD patients when compared with the controls (53, 54). Another evidence comes from the presence of microglial cells surrounding amyloid plaques in AD cerebral cortex, the presence of Aβ deposition in T-cells can activate microglia and reactive astrocytes in the brains of AD patients (55). Moreover, the up-regulation of APP is also associated with neuroinflammation. Inflammatory responses are strongly associated with the altered expression of microRNAs in the AD brain. In order to elucidate which microRNA is important in the production of pro-inflammatory cytokines and proteolytic enzymes in AD, mRNA targets and specific roles in brain need to be identified and established.

Several research groups have investigated the effects of microRNAs on LPS-induced neuroinflammation and Toll-like receptor 4 (TLR4)-mediated inflammation. The miR-132 is involved in multiple physiological and pathological mechanisms, such as neuronal cell development (56), synaptic plasticity (57, 58) and inflammation (59, 60). Recent studies indicate that miR-132 participates in the regulation of inflammation and is a negative regulator of the inflammatory response in PC12 (61). Interestingly, resveratrol treatment could ameliorate inflammatory response in PC-12 cells via up-regulating miR-132. Moreover, based on this report, IL-1β, IL-6, and TNF-α are proposed as the targets of miR-132. Similarly, miR-132 is down-regulated in LPS-induced inflammatory injury in neuron HT-22 cells and the overexpression of miR-132 attenuates the inflammatory response (62). TNF receptor associated factor 6 (TRAF6) linked to promote inflammation may be a direct target of miR-132. In addition, miR-206 can enhance LPS-induced inflammation and promote the release of Aβ in microglia by binding to the 3'-UTR of insulin-like growth factor 1 (IGF-1) so that IGF-1 exposure can mitigate miR-206-induced inflammation in microglia, indicating that the miR-206/IGF-1 signaling pathway may be associated with microglial inflammation in AD (63).

Persistent microglial activation is able to initiate inflammatory activity, results in neuronal damage and eventually causes AD. MiR-155 is one of the most well-studied microRNAs in AD-related neuroinflammatory events. In 3xTg AD animal model, there is a high expression level of miR-155. The up-regulation of miR-155 is simultaneously accompanied with an enhanced activation of microglia and astrocytes, thus triggering the production of inflammatory mediators. Moreover, miR-155 can also contribute to the regulation of AD through activating different T cell functions during inflammation (64). Clinical data from human AD brains indicate that miR-125b and miR-146 levels are elevated to aggravate neuroinflammation and reduce complement factor H, which is associated with the neuronal release of mR-146a and miR-155 and inflammatory spreading in the AD brain (65, 66). During investigating the significance of microRNA release in the AD brain, let-7 family has also gained extensive attention. Let-7 has been reported to be critical for maintaining microglial function in inflammation-mediated injury (67). In the studies on let-7a in LPS-treated microglial BV2 cells, let-7a level is found to be remarkably decreased; however, let-7a overexpression can reduce the production of inducible nitric oxide synthase (iNOS) and IL-6, while promoting anti-inflammatory genes at the same time in microglia (68). Consistent with this finding, let-7a can strongly inhibit the expression of inflammatory cytokines by controlling the activation of apoptosis signal-regulating kinase 1 (ASK1), thus activating anti-inflammatory cytokines such as IL-10 and Mycs in microglia (69). Meanwhile, let-7 could act as a regulator of microglial function during inflammation and be a novel target for enhancing the beneficial function of microglia in CNS disorders. In addition, the released let-7b activates the Toll-like receptor 7, thus resulting in neuronal degeneration. Besides, miR-32-5p knockdown also can ameliorate the production of inflammatory cytokines in LPS-treated microglia and dual-specificity phosphatase 5 (Dusp5) is a direct target of miR-32 (70). Similar with this, miR-204 inhibition could repress inflammation process in LPS-induced mouse microglial cell lines (N9 and BV2) via regulating Sirt1 level (71). One of previous studies has found that the loss of miR-29a disrupts the activity of neuronal navigator 3 that is involved in guidance, and is enriched in degenerating pyramidal neurons in AD (72). Thus, above microRNAs may provide potential therapeutic strategies for neuroinflammation. The pharmacological modulation of microRNAs in anti-inflammatory response can be achieved. For example, klotho at different concentrations (0.5, 1 and 2 nM) or linagliptin (50 μM) can inhibit the expression of TNF-α and then alleviate the inflammation in human peripheral blood mononuclear cells (PBMCs) of AD patients, probably by suppressing inflammatory cytokines and up-regulating miR-29a (73). Therefore, microRNAs may have the therapeutic potential of AD through attenuating neuroinflammation.

### microRNA-Mediated Aging in AD

Aging is accompanied with behavioral impairments at different degrees, including impaired learning and memory capacity. Increasing evidence suggests that numerous microRNAs are largely implicated in aging and cellular changes associated with aging. Thus, it is very important to evaluate microRNAs that affect these aging events in order to determine the roles of microRNAs in aging. Previous studies have demonstrated that the majority of microRNAs such as miR-151a-3p, miR-181a-5p, miR-1248, miR-103, miR-107, miR-128, miR-130a, miR-155, miR-24, miR-221, miR-496, and miR-1538 in serum of human are down-regulated as the extension of age (74). Interestingly, miR-1248 and miR-181a are negatively associated with the expression of IL-6 and TNF-α and positively correlated with anti-inflammatory cytokines including TGF-β and IL-10, suggesting that circulating microRNAs could be the biological markers of aging. Data from peripheral blood of AD subjects have demonstrated that miR-34a expression is remarkably up-regulated when compared to normal elderly controls (39, 75). In addition, compared with age-matched healthy control, the increased expression of miR-34a in the brain is closely associated with the severity of AD (76). Also, human and mouse SIRT1 mRNA are the targets of miR-34a, so that miR-34a expression is closely related with human longevity (77). Our research team has also found that swimming intervention with a period of 8 weeks can attenuate brain aging in D-galactose-induced AD rats. Mechanically, swimming training down-regulates miR-34a expression in AD rats, which is also confirmed by miR-34a inhibitor in SH-SY5Y cells (78). In normally aged mice, resveratrol treatment can improve learning and memory capacity through down-regulating miR-124/-134, in turn, activating CREB-BDNF signal pathway, which suggests that a resveratrol-rich diet may be beneficial for preserving cognitive function in aged individuals (79). Up to date, the contribution of microRNAs to age and/or senescence-related changes in gene expression is not completely clear. For example, miR-17, miR-19b, miR-20a, and miR-106a are significantly down-regulated in various human aging model systems (80). Meanwhile, the decrease in these microRNAs is correlated with the increased transcript levels of CDK inhibitor p21/CDKN1A. Similar to this, a series of microRNAs such as miR-9, miR-19a, miR-135a, miR-15b, miR-16, miR-214, and miR-141 are reported to be associated with aging (81, 82). These findings indicate that microRNAs can be the novel markers of cell aging in humans.

### Dysfunctional Autophagy and Autolysosomal Proteolysis Mediated by microRNAs in AD

Autophagy involved in the degradation of long-lived proteins, cytosolic components, or damaged organelles is essential for the survival of mature neurons (83–85). Multiple studies have demonstrated that the induction of autophagy plays a neuroprotective role; on the contrary, deficient autophagy or impaired autophagic flux can result in neurological damage in most neurological disorders (86–88). The regulation of Aβ and Tau is critically affected by autophagy. For example, phosphorylated Tau in neurons is mainly removed by normal autophagy, and autophagy activation or enhancement can effectively promote the clearance of Tau (89). In addition, loss-of-function mutations in several genes with autophagy-related function such as Becn1/VPS30/ATG6 (90), Atg7 (91), and Atg5 (92) can result in dysfunctional autophagy and increased accumulation of disordered and aggregated proteins such as Aβ and Tau in AD, indicating that autophagy failure has become an important therapeutic target for AD and regulating autophagy by exogenous means may become a new strategy for AD treatment. Beclin1 plays a significant role in autophagy. The studies from cultured neurons and transgenic mice have verified that the deficiency of Beclin1 can provoke the deposition of Aβ; however, Beclin1 overexpression can mitigate the accumulation of Aβ (93). Moreover, the induction of autophagy via the administration of a lentiviral vector expressing Beclin1 can decrease both intracellular and extracellular Aβ pathology in APP transgenic mice (90). Rapamycin, as an autophagy inducer, can attenuate Aβ accumulation and inhibit Tau phosphorylation in AD mouse models (94). Similarly, autophagy-related genes including LC3-II/LC3-I, Beclin1, Atg7, and autophagic influx are markedly decreased in D-galactose-induced AD rat models when compared with the control; however, 8-week swimming training (autophagy mimics) alleviates cognitive function defects via restoring autophagy in an AD rat model (78). Above findings reveal the reasonable proposition that the induction of autophagy has potential therapeutic benefits in AD. However, considering the functional status of autophagy in AD is context-dependent and complex, controversial data about the applicability of inducing autophagy as a general treatment strategy for AD are also exist. For example, autophagy inhibition has been reported to mitigate Aβ42-induced cell death (95, 96), thus, it appears that the time of intervention for inducing autophagy during the progression of these neurodegenerative diseases should be considered during implementing autophagy induction as a therapeutic approach. As illustrated from one of previous studies, increasing induction of autophagy prior to the development of AD-like pathology in 3×Tg-AD mice can reduce the levels of soluble Aβ, Tau and amyloid plaques, whereas the induction after the formation of mature plaques and tangles has no effect on AD-like pathology or cognitive deficits (97).

In addition to the defects of autophagy at the early stages, autolysosomal proteolysis is significantly impaired in AD and its defect is one of the key pathogenic factors in AD (98, 99); thus, selectively enhancing lysosomal activity by genetic ablation of cystatin B to enhance the clearance of autophagic substrates and ameliorate amyloid pathology and memory deficits in TgCRND8 AD mouse models (100). The recovery of autophagic flux is crucial for reversing spatial learning and cognitive deficit. It has been reported that autophagic flux of AD patients is impaired, and autophagic sequestration is stimulated in AD patients at the early stage, while lysosomal clearance is progressively declined and autophagic flux is gradually hindered due to the lack of the substrate clearance (101). Similarly, increased autophagic flux with daily intra-peritoneal injection of pimozide in AD mice decreases the aggregation of Tau through the mTOR-independent AMPK-ULK1 axis (102). Additionally, the treatment with rapamycin and an anti-epileptic drug carbamazepine can alleviate cognitive impairment and Aβ neuropathology in APP/PS1 transgenic mouse model through restoring normal autophagy (103). These observations support the disruption of substrate proteolysis within autolysosomes as the principal mechanism of dysfunctional autophagy in AD.

In recent years, accumulating evidence suggests that microRNAs play an important role for the regulation of autophagy in brain tissue of AD patients. In addition to the correlation with aging, miR-34 is also linked to autophagy and longevity in several species. The decreased miR-34 level is detected in long-lived dietary-restricted mice. In human cells, miR-34 can target Bcl-2, thereby directly inhibiting autophagy-related BECN1/VPS30 complex (104). According to the reports (105), miR-214-3p is also down-regulated in hippocampal neurons of SAMP8 mice and cerebrospinal fluid from sporadic Alzheimer's disease. The treatment with miR-214-3p for SAMP8 mice improves behavioral performance and attenuates neuronal apoptosis. Another study (106) has also demonstrated that miR-214-3 is down-regulated in patients and model animals with AD. It negatively regulates autophagy, thus exerting its neuroprotective effects and Atg12 is a direct target of miR-214-3p in neurons. MiR-299-5p is also a potent autophagy regulator, and the decreased level of miR-299-5p is also reported in hippocampal tissue of APPswe/PS1dE9 mice and cerebrospinal fluid of AD patients. Strikingly, the overexpression of miR-299-5p promotes cognitive impairment of APPswe/PS1dE9 mice via modulating autophagy and apoptosis by targeting Atg5 (107). Similarly, miR-376a, miR-376b, and miR-181a can prevent starvation-induced autophagy in human cell lines by blocking the expression of Beclin1, Atg4c, or Atg5 (108). In addition, the expression of miR-132/212 in AD brain is also observable, and miR-132/212 is down-regulated in AD (109). Mechanistically, Atg9a and Atg5-12 are its targets. Besides, recent studies have found that autophagy is also regulated by miR-30d and miR-101 through inhibiting Beclin1 and Atg4d expression, which may be a new mechanism for AD (110, 111). The induction of autophagy by pharmacological administration such as resveratrol, osthole and ampelopsin has been proved to effectively reduce neuronal aggregates and alleviate the progression of neurological symptoms in several mouse models with AD through activating microRNA-mediated autophagy (112, 113).

## Remarks and Future Directions

**Figure 1:**
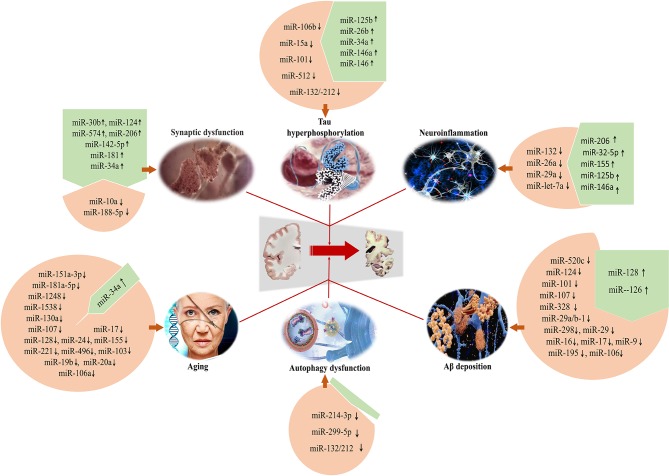
Specific microRNAs involved in the development of AD for regulating Aβ deposition, Tau hyperphosphorylation, synaptic dysfunction, neuroinflammation, and autophagic dysfunction. Meanwhile, microRNAs can be considered as the preventive and therapeutic targets to develop novel and effective intervention strategies for AD.

The deposition of Aβ, intracellular aggregation of hyperphosphorylated Tau protein, the loss of synapses, neuroinflamamiton and autophagic dysfunction, as well as aging reveal the critical roles in the pathogenesis of AD, which is associated with the dysfunctional regulation of a series of microRNAs (Figure 1). Given the large number of microRNAs involved in AD, the analysis of microRNAs in body fluids is a relatively simple procedure when compared with structural magnetic resonance imaging (MRI) and molecular neuroimaging ith positron emission tomography (PET), and microRNAs appear to be promising. A more complete understanding of the regulatory roles of specific microRNAs in AD will be helpful for the development of therapeutic strategies. Therefore, microRNAs as diagnostic and therapeutic agents in AD should be extensively explored and applied in the future. However, possible limitations of microRNAs including the induction of autophagy at suitable stages need to be further explored and clarified.

## Author Contributions

NC and XK have designed the project and executed the manuscript writting. XK and DC have participated the literature collection and draft writting. NC have conducted the editing and final reviewing of the manuscript.

## Conflict of Interest

The authors declare that the research was conducted in the absence of any commercial or financial relationships that could be construed as a potential conflict of interest.
